# Characterization of the Adaptive Amoxicillin Resistance of *Lactobacillus casei* Zhang by Proteomic Analysis

**DOI:** 10.3389/fmicb.2018.00292

**Published:** 2018-02-20

**Authors:** Jicheng Wang, Huiling Guo, Chenxia Cao, Wei Zhao, Lai-Yu Kwok, Heping Zhang, Wenyi Zhang

**Affiliations:** Key Laboratory of Dairy Biotechnology and Engineering, Ministry of Education, Key Laboratory of Dairy Products Processing, Ministry of Agriculture, Inner Mongolia Agricultural University, Hohhot, China

**Keywords:** *Lactobacillus casei* Zhang, proteomic analysis, amoxicillin, adaptive evolution, stress resistance

## Abstract

Amoxicillin is one of the most commonly prescribed antibiotics for bacterial infections and gastrointestinal disorders. To investigate the adaptation of *Lactobacillus* (*L*.) *casei* Zhang to amoxicillin stress, an iTRAQ-based comparative proteomic analysis was performed to compare the protein profiles between the parental *L*. *casei* Zhang and its amoxicillin-resistant descendent strains. Our results revealed a significant increase in the relative expression of 38 proteins (>2.0-folds, *P* < 0.05), while the relative expression of 34 proteins significantly decreased (<−2.0-folds, *P* < 0.05). The amoxicillin-resistant descendent strain exhibited marked alterations in carbohydrate and amino acid metabolism. Moreover, certain components involving in membrane metabolism were activated. The differences in the proteomic profiles between the two strains might explain the enhanced stress resistance of the adapted bacteria.

## Introduction

Antibiotic resistance is a global health problem. Particularly, many antibiotics have become less effective due to rapid bacterial adaptive evolution facilitated by the frequent use of antibiotics in medicine and agriculture (Arias and Murray, 2015). Meanwhile, the increased use of antibiotics can also introduce a selective pressure which leads to the development of multi-resistance characteristics in some of the bacterial populations (Chen and Jiang, 2014). Owing to significant clinical concerns, many previous studies have investigated the relationship between antibiotic resistance and genome stability of pathogenic bacteria, especially when environmental antibiotic selective pressure is present (Andersson, 2006). Meanwhile, it is crucial to develop new strategies to prevent and control the spread of antibiotic resistance (Normark and Normark, 2002). One successful strategy is to minimize the use of antibiotics by including alternative and/or adjunct treatments for certain diseases (Schultz and Haas, 2011). For example, the combined use of probiotics and antibiotics was clinically effective in treating gastrointestinal disorders (Wright et al., 2015). Thus, there is growing interest in applying probiotics to improve human health and in clinical practice (Reid, 2017).

One concern, however, is the potential risk of evolutionary adaptation of probiotics toward antibiotic resistance after prolonged drug exposure (Perreten et al., 1997). Since most published studies have focused only on pathogenic bacteria, there are insufficient data to assess the safe use of probiotic bacteria in clinical practice. Although horizontal gene transfer is a major mechanism that had shaped the antibiotic resistance pattern of probiotics bacteria in evolution, genome point mutations cannot be neglected (Woodford and Ellington, 2007). Some lactobacilli strains have been shown to gain antibiotic resistance via point mutations (Curragh and Colllns, 1992). On the other hand, by a whole-genome resequencing approach, our earlier work monitored the genetic changes of *Lactobacillus casei* Zhang during long-term culture in an antibiotics-containing medium; and we found that, unlike pathogenic bacteria, the accumulation of *de novo* mutations occurred only initially but not after an extended period of antibiotics selection (Wang et al., 2017). However, mechanistic changes occurring at the functional level remain uncharacterized.

In a previous long-term propagation experiment, our laboratory isolated an *L. casei* Zhang descendent (*L. casei* Zhang-A-600) that had elevated resistance to amoxicillin (Wang et al., 2017). Amoxicillin is one of the most commonly prescribed antibiotics used for treating bacterial infections and gastrointestinal disorders (Kabbani et al., 2017; Zerbetto De Palma et al., 2017). It kills bacteria by inhibiting the process of cell wall synthesis. Although amoxicillin resistance is not yet considered as a serious clinical concern, several amoxicillin-tolerant strains have been isolated from gastric biopsy specimens of patients (van Zwet et al., 1998); thus, its clinical significance should not be neglected. The present work hypothesized that the amoxicillin-resistant descendent adapted to antibiotics stress via modulating cellular protein expression. Comparative proteomics analysis is an efficient tool to reveal functional differences between wild-type and mutant cells or between cells cultivated under different conditions (Wang et al., 2013). Thus, an iTRAQ-based proteomic analysis was performed to elucidate the resistant phenotype of the descendent at a global protein expression level.

## Materials and methods

### Bacterial isolates and growth

The *L. casei* Zhang descendent strain (*L. casei* Zhang-A-600) was more resistant to amoxicillin resistance with a minimum inhibitory concentration (MIC) of 8 μg/mL (vs. 2 μg/mL for the parental strain; Wang et al., 2017). The resistant strain was isolated from long-term propagation of the parental strain in lactic acid bacteria (LAB) susceptibility test medium broth (LSM), consisting of 90% Iso-sensitest medium (IST; OXOID, CM0473) and 10% MRS (Klare et al., 2005), supplemented with 0.5 μg/mL amoxicillin (Wang et al., 2017). At this antibiotics concentration, the bacterial growth was suppressed by 50%. The growth of bacteria (optical density, pH, and viable counts) was monitored every 2 h (from 0 to 30 h of cultivation). All experiments were performed in triplicate.

### Sample preparation

To minimize the interfering effect between the antimicrobial compound and the growth medium components (Klare et al., 2005), the minimal growth medium, LSM, was chosen for the current study. For proteomic analysis, the parental and descendent *L. casei* Zhang cells were collected after 24 h of cultivation in amoxicillin-containing LSM (0.5 μg/mL of amoxicillin). In each case, 4 biological replicates of samples were prepared. The culture conditions used here were the same as our previous study which aimed to characterize the adaptation of the resistant strain at the genomic level (Wang et al., 2017). Briefly, the bacterial cells were pelleted by centrifugation and washed 4 times with phosphate buffered saline (PBS). One milliliter of lysis buffer (7 M urea, 4% SDS, and 1x protease inhibitor cocktail) was added to each sample. The mixtures were then sonicated on ice and spun at 13,000 rpm for 10 min at 4°C. The supernatant of each sample was separately collected.

### Protein digestion and iTRAQ labeling

The protein concentration of the supernatants was estimated by the bicinchoninic acid protein assay. One hundred microgram of protein of each sample was adjusted to a final volume of 100 μL with 100 mM triethylammonium bicarbonate (TEAB), followed by adding 5 μL of 200 mM DTT and incubating at 55°C for 1 h. Afterwards, 5 μL of iodoacetamide (375 mM) was added to each sample, followed by 30 min incubation in dark at room temperature. Then the protein was precipitated with ice-cold acetone and redissolved in TEAB (20 μL). Proteins were digested with sequence-grade modified trypsin (Promega, Madison, WI) and labeled using the iTRAQ reagents kit. The labeled samples were combined, desalted (Sep-Pak C18 SPE column, Waters, Milford, MA), and vacuum dried.

### High pH reverse phase separation

Phase separation was performed as described by Gilar with some modifications (Gilar et al., 2005). Briefly, the peptide mixture was redissolved in buffer A (buffer A: 10 mM ammonium formate in water, pH 10.0, adjusted with ammonium hydroxide). The dissolved peptide mixtures were then fractionated by high pH separation using the Aquity UPLC system (Waters Corporation, Milford, MA) connected to a reverse phase column (BEH C18 column, 2.1 × 150 mm, 1.7 μm, 300 Å, Waters Corporation, Milford, MA). A linear gradient, starting from 0% B to 45% B in 35 min (B: 10 mM ammonium formate in 90% ACN, pH 10.0, adjusted with ammonium hydroxide), was used in the high pH separation. The column flow rate and temperature were maintained at 250 μL/min and 45°C, respectively. Sixteen fractions were separately collected and dried in a vacuum concentrator.

### Low pH Nano-HPLC-MS/MS analysis

The fractions were redissolved in a solvent composed of solvents C and D (C: 0.1% formic acid in water; D: 0.1% formic acid in ACN), separated by nano LC and analyzed by on-line electrospray tandem mass spectrometry. The experiments were performed on a Nano Aquity UPLC system (Waters Corporation, Milford, MA) connected to a quadrupole-Orbitrap mass spectrometer (Q-Exactive) (Thermo Fisher Scientific, Bremen, Germany) with an online nano-electrospray ion source. Eight microliters of each peptide sample were loaded onto the trap column (Thermo Scientific Acclaim PepMap C18, 100 μm × 2 cm), with a flow of 10 μl/min for 3 min, to be separated on a 75 μm × 25 cm Acclaim PepMap C18 analytical column. A linear gradient, from 5% D to 30% D in 95 min, was used. The column was re-equilibrated at initial conditions for 15 min. The column flow rate and temperature were maintained at 300 μL/min and 45°C, respectively. An electrospray voltage of 2.0 kV was applied to the mass spectrometer inlet.

The Q-Exactive mass spectrometer was operated in the data-dependent mode, switching automatically between MS and MS/MS acquisition. Survey full-scan MS spectra (m/z 350–1,600) of mass resolution of 70K were acquired, followed by 15 sequential higher-energy collisional dissociation (HCD) MS/MS scans of 17.5K resolution. In all cases, one 30-s dynamic exclusion micro-scan was recorded. The MS/MS fixed first mass was set to 100.

### Database searching

Tandem mass spectra were extracted by ProteoWizard (version 3.0.5126; Thermo Fisher Scientific) using the Proteome Discoverer software (version 1.4.0.288; Thermo Fisher Scientific). All MS/MS samples were analyzed using Mascot (version 2.3; Matrix Science, London, UK), which was used to search the NCBI database (Taxonomy: *Lactobacillus casei* Zhang, 2804 entries; trypsin digestion; 0.050 Da fragment ion mass tolerance and 10.0 PPM parent ion tolerance). Moreover, in the Mascot search, cysteine carbamidomethylation and iTRAQ 8plex of lysine and the n-terminus were opted as fixed modifications, while methionine oxidation and iTRAQ 8plex of tyrosine were specified as variable modifications.

### Quantitative data analysis

Statistical analyses were performed following the recommendations of Predrag Radivojac and Olga Vitek (Radivojac and Vitek, 2012). The percolator algorithm of <1% was used to control the false discovery rate. Only unique peptides were quantified. Experimental biases were corrected by normalization with the protein median. The minimum number of observed proteins was 1000. Statistical analysis was performed under the R environment (Student's *t*-tests, *p* < 0.05 was considered statistically significant). A cut-off level of 2.0-fold change was applied to select differentially expressed proteins; and only those showing a consistent expression change in all 4 biological replicates were considered as differentially expressed proteins. They were functionally assigned by the clusters of orthologous genes (COGs) and the Kyoto Encyclopedia of Genes and Genomes databases (Tatusov et al., 1997).

### Construction and analysis of gene disruption mutants

Two genes, LCAZH_0490 and LCAZH_0521, were selected as target candidates to be genetically disrupted. They putatively encoded an OmpR family DNA-binding response regulator and a surface-associated protein, respectively. The plasmids and primers used for constructing the gene disruption mutants are listed in Table 1.

**Table 1:** Strains, plasmids, and primers used in this study.

**Strain, plasmids, and primers**	**Description or primer sequence^a^**	**Reference or source**
**STRAINS**		
*E. coli* DH5α	Cloning host	This study
*L. casei* Zhang	Isolated from home-made koumiss in Inner Mongolia, China	This study
*L. casei* Zhang-A-600	*L. casei* Zhang propagated in LSM broth containing amoxicillin 0.5 μg/mL for 3 months	This study
*L. casei* Zhang-A-600-0490::*lox66*-P32-*cat-lox71*	Derivative of *L. casei* Zhang-A-600 containing a *lox66*-P32-*cat-lox71* replacement of LCAZH_0490	This study
*L. casei* Zhang-A-600-0521::*lox66*-P32-*cat-lox71*	Derivative of *L. casei* Zhang-A-600 containing a *lox66*-P32-*cat-lox71* replacement of LCAZH_0521	This study
*L. case*i Zhang-A-600-Δ0490	Derivative of *L. casei* Zhang-A-600-0490::*lox66*-P32-*cat-lox71* containing a *lox72* replacement of LCAZH_0490	This study
*L. case*i Zhang-A-600-Δ0521	Derivative of *L. casei* Zhang-A-600-0521::*lox66*-P32-*cat-lox71* containing a *lox72* replacement of LCAZH_0521	This study
**PLASMIDS**		
pNZ5319	Cm^r^Em^r^; containing *lox66*-P32-*cat-lox71* cassette for multiple gene replacement in gram-positive bacteria	Lambert et al., 2007
pNZ5319-0490Up-Down	Cm^r^Em^r^; pNZ5319 derivative containing homologous regions up- and downstream of LCAZH_0490	This study
pNZ5319-0521Up-Down	Cm^r^Em^r^; pNZ5319 derivative containing homologous regions up- and downstream of LCAZH_0521	This study
pMSPcre	Em^r^; expression of *cre*	unpublished
**PRIMERS**		
0490upF	5′-CCG*CTCGAG*TTTCGGGTTGTGGTGGTA-3′	This study
0490upR	5′-AGCTTT*GTTTAAAC*TTTCTTTGTTATGCCTACTG-3′	This study
0490downF	5′-GGGTTT*GAGCTC*ATAAATGGACAAGCTGAAGCGACGC-3′	This study
0490downR	5′-GA*AGATCT*GCGTTTGGTGAGCCCTTC-3′	This study
0521upF	5′-CCG*CTCGAG*TTGAGTTCCTCCAGTGTT-3′	This study
0521upR	5′-AGCTTT*GTTTAAAC*TGATTGTTAGCGGTTTCG-3′	This study
0521downF	5′-GGGTTT*GAGCTC*CTAAACTAAGGGGCAGCGGTCATTC-3′	This study
0521downR	5′-GA*AGATCT*TGTTTCGTCTCATCGGTCT-3′	This study
85	5′-GTTTTTTTCTAGTCCAAGCTCACA-3′	Lambert et al., 2007
87	5′-GCCGACTGTACTTTCGGATCCT-3′	Lambert et al., 2007
CatF	5′-TCAAATACAGCTTTTAGAACTGG-3′	Lambert et al., 2007
CatR	5′-ACCATCAAAAATTGTATAAAGTGGC-3′	Lambert et al., 2007
EryintF	5′-CGATACCGTTTACGAAATTGG-3′	Lambert et al., 2007
EryintR	5′-CTTGCTCATAAGTAACGGTAC-3′	Lambert et al., 2007

a *The restriction sites in the primer sequences are underlined*.

The gene disruption mutants were constructed using a cre-lox-based system (Lambert et al., 2007). To disrupt the LCAZH_0490 gene, the upstream and downstream flanking regions of the LCAZH_0490 gene were amplified by PCR by two primer pairs (0490upF and 0490upR; 0490downF and 0490downR) using the genomic DNA of *L. casei* Zhang-A-600 as template. These fragments were then cloned between the *Xho* I or *Pme* I and *Ecol*53 KI or *Bgl* II restriction sites of the suicide vector pNZ5319 to create the recombinant mutagenesis vector, pNZ5319-0490 Up-Down, which was introduced into *L. casei* Zhang-A-600 by electroporation. Colonies harboring the anticipated inserts in the desired orientation were identified by the combined use of the primers 85, 87, and an insert-specific primer. Chloramphenicol-resistant transformants were selected and replica plated to check for erythromycin-sensitive clones. Candidate double-crossover mutant clones were first analyzed by PCR (with the primer pairs, catR and catF, EryintF, and EryintR), followed by verifying the correct integration of the 0490::lox66-P32-cat-lox71 cassette into the genome using the primer combination of 0490upF or catR and catF or 0490downR. In order to excise the P32-cat selectable marker cassette, the cre expression plasmid, pMSPcre, was transformed into the 0490::lox66-P32-cat-lox71 gene replacement mutant. Erythromycin-resistant and chloramphenicol-sensitive colonies were selected by another round of replica plating. The cre-mediated recombination and correct excision of the P32-cat cassette were confirmed by PCR using primers spanning the recombination locus (0490upF and 0490downR). The pMSPcre vector was cured from *L. casei* Zhang-A-600 Δ0490 colonies by growth without erythromycin selection pressure. This plasmid was constructed from pMSP3535 (provided by Professor Jian Kong, Shandong University, unpublished). Similar procedures were used to inactivate the LCAZH_0521 gene in *L. casei* Zhang-A-600.

The amoxicillin-resistant phenotype of the gene disrupted mutants were evaluated by determining their MICs of amoxicillin (Guo et al., 2017). Moreover, the OD value at the time of observing the MIC by witness was determined to quantify the growth performance of the wild-type and mutant strains. The Student's *t*-test at a confidence level of 0.05 was used to evaluate any difference in the growth performance between different strains.

### Nucleotide sequence accession number

The gene disruption plasmids were designed and constructed based on the genome sequence of *L. casei* Zhang (GenBank database accession number: CP001084.2). The sequences of gene disruption regions of the mutant strains, Zhang-A-600-delta-0490 and Zhang-A-600-delta-0521, have been deposited to the GenBank database under the accession numbers MG021089 and MG021090.

## Results

### Growth of *L. casei* Zhang-A-600 and the parental strains

**Figure 1:**
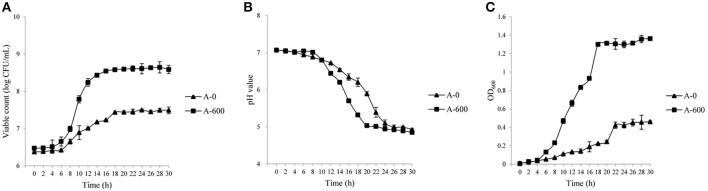
Growth of *Lactobacillus casei* strains in lactic acid bacteria susceptibility test medium broth (LSM) supplemented with amoxicillin. Changes in the viable counts **(A)**, pH **(B)**, and OD_600_ **(C)** were monitored over 30 h. The parental and the amoxicillin-resistant (Zhang-A-600) strains are represented by “A-0” and “A-600,” respectively. Error bars represent standard deviation.

The growth characteristics (viable counts, pH, and OD values) of the parental *L. casei* Zhang strain and the amoxicillin-resistant Zhang-A-600 descendent were monitored. The growth performance of *L. casei* Zhang-A-600 was better than that of the parental strain when amoxicillin was present in the culture medium (Figures 1A–C). Both the maximum viable count and cell density of *L. casei* Zhang-A-600 were significantly higher (*P* < 0.05) than that of the parental strain, which were 6.3 × 10^8^ CFU/mL and 1.36, respectively. The *L. casei* Zhang-A-600 culture entered stationary phase at 12 h (vs. 10 h for the parental strain; Figure 1A). Moreover, the pH of the *L. casei* Zhang-A-600 culture dropped much faster than that of the parental strain (Figure 1B).

### Differentially expressed proteins identified in *L. casei* Zhang-A-600

When *L. casei* Zhang-A-600 was grown in the presence of amoxicillin, the expression of 38 proteins significantly increased (>2.0-folds, *P* < 0.05) compared with the parental cells (Table 2). Most of these proteins could be assigned to specific COG functional categories.

**Table 2:** Up-regulated *Lactobacillus casei* Zhang-A-600 proteins in comparison with the parental strain.

**Gene**	**Function**	**COG^a^**	**Fold change**	***P*-value**
LCAZH_0295	PTS system cellobiose-specific transporter subunit IIC	[G]	10.95	<0.05
LCAZH_0393	PTS system fructose-specific transporter subunit IIABC	[G]	3.83	<0.05
LCAZH_0394	glycosyl hydrolase	[G]	2.69	<0.05
LCAZH_2653	trehalose-6-phosphate hydrolase	[G]	13.2	<0.05
LCAZH_2725	transaldolase	[G]	3.12	<0.05
LCAZH_0339	oligopeptide ABC transporter periplasmic protein	[E]	2.03	<0.05
LCAZH_0419	amino acid ABC transporter substrate-binding protein	[ET]	2.42	<0.05
LCAZH_1957	amino acid ABC transporter permease	[E]	7.98	<0.05
LCAZH_1958	amino acid ABC transporter permease	[E]	3.69	<0.05
LCAZH_1959	amino acid ABC transporter substrate-binding protein	[ET]	7.47	<0.05
LCAZH_1960	polar amino acid ABC transporter ATPase	[E]	8.63	<0.05
LCAZH_0682	malolactic enzyme	[C]	3.69	<0.05
LCAZH_2132	acetate kinase	[C]	2.87	<0.05
LCAZH_2374	Old Yellow Enzyme family NADH:flavin oxidoreductase	[C]	4.43	<0.05
LCAZH_2075	ACP S-malonyltransferase	[I]	2.04	<0.05
LCAZH_0172	transcriptional regulator	[K]	2.27	<0.05
LCAZH_0502	transcriptional regulator	[K]	2.68	<0.05
LCAZH_0490	OmpR family DNA-binding response regulator	[TK]	3.89	<0.05
LCAZH_0491	signal transduction histidine kinase	[T]	2.65	<0.05
LCAZH_0447	conjugated bile salt hydrolase-like amidase	[M]	10.19	<0.05
LCAZH_0562	nucleoside-diphosphate-sugar epimerase	[MG]	3.93	<0.05
LCAZH_2067	cyclopropane fatty acid synthase-like methyltransferase	[M]	3.15	<0.05
LCAZH_0279	ADP-ribosylglycohydrolase	[O]	2.55	<0.05
LCAZH_1136	multidrug ABC transporter ATPase/permease	[V]	2.09	<0.05
LCAZH_2155	multidrug ABC transporter ATPase/permease	[V]	2.52	<0.05
LCAZH_0294	alpha/beta hydrolase	[R]	3.58	<0.05
LCAZH_1865	dinucleotide-binding enzyme	[R]	3.31	<0.05
LCAZH_2372	oxidoreductase	[R]	4.49	<0.05
LCAZH_0186	hypothetical protein	-	2.7	<0.05
LCAZH_0273	cell wall-associated hydrolase	-	2.09	<0.05
LCAZH_0444	hypothetical protein	[S]	2.05	<0.05
LCAZH_0458	XRE family transcriptional regulator	-	4.17	<0.05
LCAZH_0521	putative surface-associated protein	-	5.93	<0.05
LCAZH_1898	hypothetical protein	-	5.53	<0.05
LCAZH_2301	hypothetical protein	[S]	11.5	<0.05
LCAZH_2317	hypothetical protein	[S]	3.47	<0.05
LCAZH_2327	hypothetical protein	-	4.33	<0.05
LCAZH_2435	hypothetical protein	-	16.11	<0.05

a *COG functional categories: [G], Carbohydrate transport and metabolism; [E], Amino acid transport and metabolism; [T], Signal transduction mechanisms; [C], Energy production and conversion; [I], Lipid transport and metabolism; [K], Transcription; [M], Cell wall/membrane/envelope biogenesis; [F], Nucleotide transport and metabolism; [H], Coenzyme transport and metabolism; [O], Post-translational modification, protein turnover, chaperones; [R], General function prediction only; [S], Function unknown; [V], Defense mechanisms*.

Six of the highly expressed proteins (15.8%) were involved in amino acid transport and metabolism (E), including a transporter component for oligopeptides (LCAZH_0339), an amino acid ABC transporter substrate-binding protein (LCAZH_0419), as well as a set of proteins responsible for amino acid transport (LCAZH_1957–LCAZH_1960). Five other highly expressed proteins (12.8%) were associated with carbohydrate transport and metabolism (G), namely the PTS system cellobiose-specific transporter subunit IIC (LCAZH_0295), the PTS system fructose-specific transporter subunit IIABC (LCAZH_0393), a trehalose-6-phosphate hydrolase (LCAZH_2653), and a transaldolase (LCAZH_2725).

Some of the highly expressed proteins fell into the COG classes T and M, which were connected with cellular stress response. These included the OmpR family DNA-binding response regulator (LCAZH_0490), a signal transduction histidine kinase (LCAZH_0491), a conjugated bile salt hydrolase-like amidase (LCAZH_0447), a nucleoside-diphosphate-sugar epimerase (LCAZH_0562), and a cyclopropane fatty acid synthase-like methyltransferase (LCAZH_2067). The functions of several other differentially expressed proteins were unknown.

In contrast to the spectrum of highly expressed proteins, the majority of the lowly expressed proteins (35.3%) belonged to the COG class G (Table 3). Among them, 4 putative proteins, namely the PTS system cellobiose-specific transporter subunits IIA and IIB (LCAZH_2637, LCAZH_2638), a triosephosphate isomerase (LCAZH_2697), and a fructose/tagatose bisphosphate aldolase (LCAZH_2698), were encoded by genes located within an operon-like structure in the *L. casei* Zhang genome. Two other lowly expressed proteins were assigned to the COG class V, i.e., the antimicrobial peptide ABC transporter permease and ATPase (LCAZH_1927, LCAZH_1928).

**Table 3:** Down-regulated *Lactobacillus casei* Zhang-A-600 proteins in comparison with the parental strain.

**Gene**	**Function**	**COG^a^**	**Fold change**	***P*-value**
LCAZH_0264	H^+^/gluconate symporter-like permease	[GE]	−4.23	<0.05
LCAZH_0355	ribose ABC transporter auxiliary component	[G]	−2.03	<0.05
LCAZH_2151	beta-glucosidase/6-phospho-beta-glucosidase/beta-galactosidase	[G]	−3.43	<0.05
LCAZH_2637	PTS system cellobiose-specific transporter subunit IIA	[G]	−5.94	<0.05
LCAZH_2638	PTS system cellobiose-specific transporter subunit IIB	[G]	−2.57	<0.05
LCAZH_2642	alpha-mannosidase	[G]	−3.77	<0.05
LCAZH_2645	hypothetical protein	[G]	−3.52	<0.05
LCAZH_2697	triosephosphate isomerase	[G]	−2.07	<0.05
LCAZH_2698	fructose/tagatose bisphosphate aldolase	[G]	−2.63	<0.05
LCAZH_2701	PTS system galacitol-specific transporter subunit IIA	[GT]	−2.08	<0.05
LCAZH_2968	2-dehydro-3-deoxygluconokinase	[G]	−2.96	<0.05
LCAZH_0739	D-alanyl carrier protein	[IQ]	−2	<0.05
LCAZH_2351	response regulator of the LytR/AlgR family	[KT]	−6.6	<0.05
LCAZH_2640	transcriptional regulator/sugar kinase	[KG]	−2.11	<0.05
LCAZH_0738	D-alanyl transfer protein	[M]	−2.13	<0.05
LCAZH_0498	membrane associated subtilisin-like serine protease	[O]	−2.22	<0.05
LCAZH_1927	antimicrobial peptide ABC transporter permease	[V]	−2.54	<0.05
LCAZH_1928	antimicrobial peptide ABC transporter ATPase	[V]	−2.86	<0.05
LCAZH_0426	short-chain alcohol dehydrogenase	[R]	−2.6	<0.05
LCAZH_0572	alpha/beta hydrolase	[R]	−2.03	<0.05
LCAZH_0041	hypothetical protein	-	−2.05	<0.05
LCAZH_0094	hypothetical protein	-	−3.22	<0.05
LCAZH_0540	hypothetical protein	-	−6.04	<0.05
LCAZH_0543	hypothetical protein	-	−2.64	<0.05
LCAZH_1179	XRE family transcriptional regulator	-	−36.73	<0.05
LCAZH_1464	hypothetical protein	-	−2.41	<0.05
LCAZH_1498	hypothetical protein	-	−3.5	<0.05
LCAZH_1530	hypothetical protein	-	−2.39	<0.05
LCAZH_2158	hypothetical protein	-	−2	<0.05
LCAZH_2238	lysyl-tRNA synthetase	[S]	−2.06	<0.05
LCAZH_2381	hypothetical protein	-	−2.87	<0.05
LCAZH_2589	hypothetical protein	[S]	−2.06	<0.05
LCAZH_2704	hypothetical protein	[S]	−6.73	<0.05
LCAZH_2722	hypothetical protein	-	−3.1	<0.05

a *COG functional categories: [E], Amino acid transport and metabolism; [G], Carbohydrate transport and metabolism; [I], Lipid transport and metabolism; [K], Transcription; [M], Cell wall/membrane/envelope biogenesis; [O], Post-translational modification, protein turnover, chaperones; [Q], Secondary metabolites biosynthesis, transport and catabolism; [R], General function prediction only; [S], Function unknown; [T], Signal transduction mechanisms; [V], Defense mechanisms*.

### Amoxicillin-resistant phenotype of the mutants

**Figure 2:**
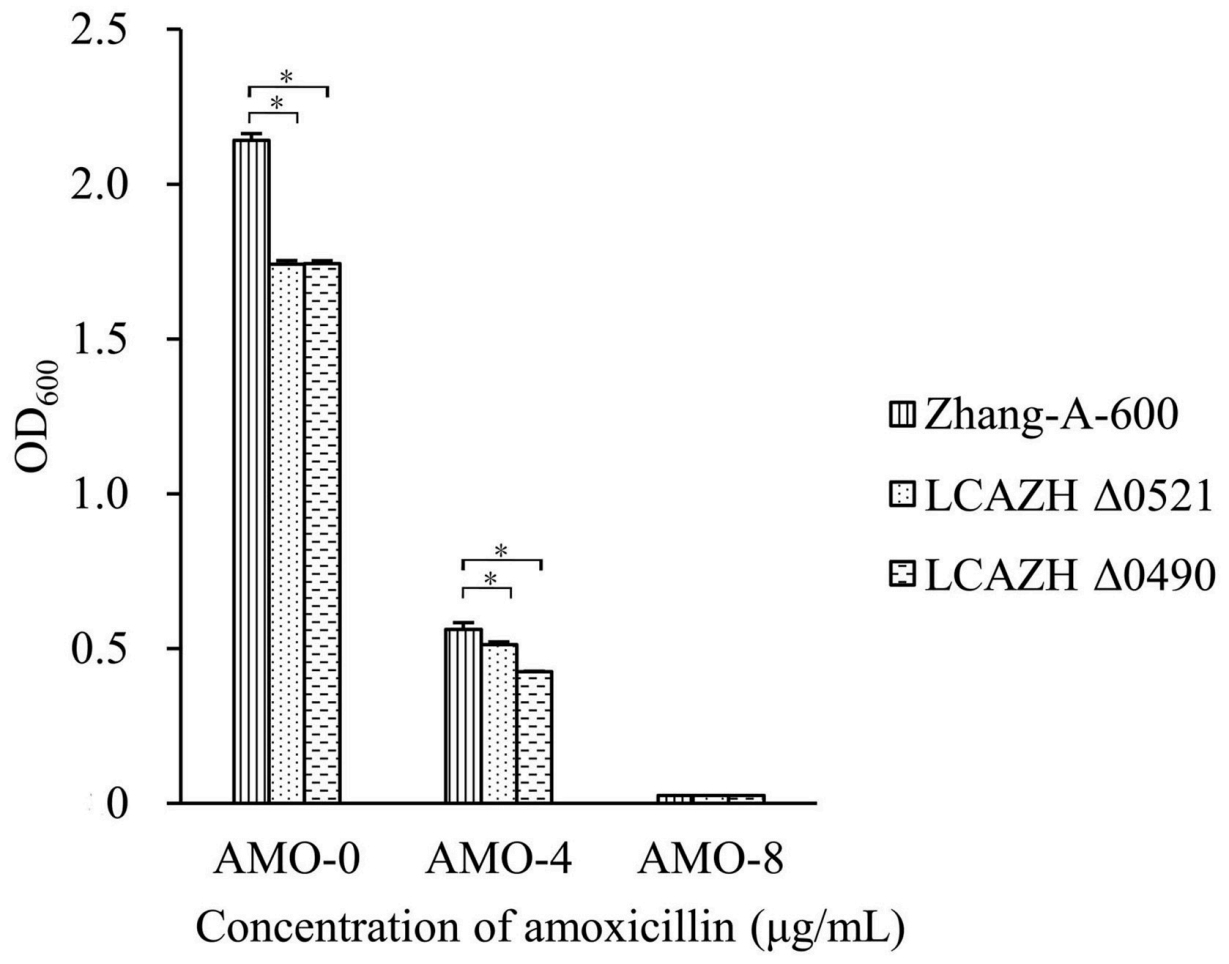
Effect of amoxicillin concentration on the OD_600_ of the culture medium of the wild-type (Zhang-A-600) and the mutants (LCAZH Δ0490 and LCAZH Δ0521). Error bars represent standard deviation. Asterisks indicate the level of statistical significance (“^*^” represents *P* < 0.05); no asterisk indicates no significance (*P* > 0.05).

The target knockout genes, LCAZH_0490 and LCAZH_0521, were selected because of their predicted molecular functions in stress response. No significant difference (*P* > 0.05) was observed in the MICs of amoxicillin between the gene disruption mutants and the wild-type, *L. casei* Zhang-A-600. However, the mutants, particularly Δ0490 that lacked the response regulator, grew slower than *L. casei* Zhang-A-600 in the presence of amoxicillin. As shown in Figure 2, the OD of the *L. casei* Zhang-A-600 culture was 1.31-fold higher (*P* < 0.05) than that of the mutant Δ0490 cultivated in LSM with 4 μg/mL amoxicillin.

## Discussion

The combined use of antibiotics and probiotics has recently been shown to improve the eradication rate for certain infections (Kafshdooz et al., 2017). However, there is yet insufficient data to assess the safe use of probiotic bacteria in clinical practice. Particularly, the antibiotics-induced adaptation responses of probiotics are not well characterized. Previously, our laboratory isolated an amoxicillin-resistant *L. casei* Zhang strain in a long-term antibiotics-driven evolution experiment. Here, we aimed to investigate the mechanisms of amoxicillin resistance of this strain using a comparative proteomics approach.

The amoxicillin-resistant isolate had altered carbohydrate metabolism. Although glucose was the main carbon source in the culture medium used in the experiment, several proteins involving in carbohydrate metabolism, including beta-glucoside metabolism (LCAZH_0295), fructose utilization (LCAZH_0393 and LCAZH_2725), and trehalose utilization (LCAZH_2653), were highly expressed compared with the parental strain. In contrast, the expression of some glycolysis- and gluconeogenesis-associated proteins (LCAZH_2697 and LCAZH_2698) decreased, reflecting an altered substrate requirement for the antibiotics-resistant strain. Interestingly, the expression of 1 component (LCAZH_0295) of the PTS system cellobiose-specific transporter subunit increased, while the relative abundance of two other components (LCAZH_2637 and LCAZH_2638) of the same transporter decreased. It is hard to explain the inconsistent changes between the individual transporter components. However, since PTS systems are involved in regulating gene expression, we speculate that the divergent expression of these components was associated with cellular protection against environmental stressors via modulation of gene expression (Nascimento et al., 2004).

The switching of carbon utilization from glucose to other substrates often happens when the growth environment turns acidic. For example, this was observed at the start of the late growth phase of *L. casei* Zhang when it was grown in cow milk and soy milk (Wang et al., 2012a,b). In addition, the growth medium used in this study was a minimal medium that was suboptimal for the growth of lactobacilli, which might have enhanced the switching of carbon utilization. The medium contained hydrolyzed casein, which could potentially serve as an alternative carbohydrate source (Williams et al., 2000). Two other proteins, an acetate kinase (LCAZH_2132) and a malolactic enzyme (LCAZH_0682), were possibly modulated by the carbon source switching as well. The former protein catalyzes the formation of acetyl phosphate from acetate and generates ATP, while the latter one catalyzes the production of L-latate and CO_2_ from L-malate via decarboxylation (Poolman et al., 1991; Puri et al., 2014). Likewise, the lack of mono-/di-carbohydrates, citrates, and amino acids in the minimal growth medium could have been another factor contributing to the carbon source switching in acidic environment. These survival strategies and acid-tolerant mechanisms are well documented (Behr et al., 2007).

Amino acids are essential for bacterial growth, and the modulation of amino acids metabolism is another strategy that helps increase bacterial stress tolerance. In order to survive, *L. casei* Zhang cells have to acquire adequate amino acids from the direct growth environment. To aid efficient acquisition of amino acids, LAB usually possess an effective proteolytic enzyme system that generally consists of multiple cell surface-associated proteinases, transport systems, and peptidases. Before any peptides or amino acids can be translocated to the cytoplasm, proteins would first need to be broken down by the bacterial proteinases (Zhang et al., 2015). Overall speaking, the expression of individual protein components of the proteolytic enzyme system was not induced except 1 oligopeptide ABC transporter periplasmic protein (LCAZH_0339), which might be necessary for oligopeptide uptake. This may suggest that the consecutive expression of the proteolytic proteins was enough to support the bacterial growth until the deceleration phase. One interesting observation regarding the amino acid metabolism was the increased expression of the protein clustered LCAZH_1957–LCAZH_1960. This is a putative transporter for polar amino acids, although its substrates are yet to be identified. This finding may suggest that polar amino acids are important in coping with environmental antibiotics stress. Moreover, the disruption of an amino acid permease-coding gene in *L. acidophilus* greatly increased the acid and bile sensitivity of the mutant (Azcarate-Peril et al., 2004).

Typically, the two component systems (TCS) consist of a sensor kinase and a response regulator; and they together play crucial roles in facilitating bacterial adaptation to environmental changes (El-Sharoud, 2005). The genome of *L. casei* encodes a relatively high number of TCS (Zhang et al., 2010), allowing cells to monitor their direct environment and respond rapidly to external stimuli, including chemical changes, acid, bile, and salt stresses (Landete et al., 2010; Alcántara et al., 2011; Revilla-Guarinos et al., 2013). Two component systems also confer adaptive antibiotic resistance to the species *Pseudomonas aeruginosa* and *Enterococcus faecalis* (Fernández et al., 2010; Hancock and Perego, 2012). In particular, the TCS of *Pseudomonas aeruginosa* activates a lipopolysaccharide modification operon that confers antibiotic resistance to the bacteria. The amoxicillin-resistant *L. casei* Zhang strain was found to have an increased expression in one TCS pair (LCAZH_0490 and LCAZH_0491). However, no significant difference was noted in the MIC of amoxicillin between the TCS-disrupted mutant and the wild-type strain, suggesting that this TCS might not contribute directly to the amoxicillin-resistant phenotype. Alternatively, the accumulated mutations in the antibiotics-resistant strain might have bypass the effect of TCS inactivation (Wang et al., 2017).

Cell surface is the interface between the bacterial cell and the environment when LAB confront situations of adversity. Acid and hypersaline stresses could cause alterations in the fatty acid metabolism of *L. casei* (Machado et al., 2004; Wu et al., 2014). Similar to our previous work, the expression of a cyclopropane fatty acid synthase-like methyltransferase (LCAZH_2067) and an ACP S-malonyltransferase (LCAZH_2075) increased in the antibiotics-resistant strain. These two proteins participate in fatty acid biosynthesis. During fatty acid biosynthesis, the cyclopropane fatty acid synthase-like methyltransferase catalyzes the addition of a methylene residue across the *cis* double bond of C16:1n_(9)_, C18:1n_(9)_, or C18:1n_(11)_ unsaturated fatty acids to form an unsaturated cyclopropane derivative; and the ACP S-malonyltransferase catalyzes the formation of malonyl-ACP (Payne et al., 2001). The activation of these enzymes could be a part of the membrane adaptation to the surrounding environment (Wang et al., 2017). Meanwhile, we also observed an increased expression in a WxL domain (IPR027994)-containing surface-associated protein (LCAZH_0521). Some WxL domain-containing proteins can interact with cell wall peptidoglycan and are responsive to stress (Brinster et al., 2007). However, no significant phenotypic change was observed in the gene disruption mutant LCAZH Δ0521. Further inspection of the genome of *L. casei* Zhang revealed two other WxL domain-containing proteins (LCAZH_0527 and LCAZH_0529); and whether they serve any compensatory role in the mutant LCAZH Δ0521 remains to be further explored.

## Conclusion

In summary, we compared the proteomes of a resistant *L. casei* Zhang strain isolated in an amoxicillin-driven evolution experiment and its parental line. The resistant descendent strain exhibited alterations in the carbohydrate, amino acid, and membrane metabolism. These metabolic adaptations might have enhanced the cell survival in response to the stressors. Interestingly, a TCS was found to be associated with the experimental evolution. However, further experiments are required to confirm its role in antibiotic resistance in probiotic bacteria.

## Author contributions

WenZ and HZ designed the study. WenZ, L-YK, and JW wrote the manuscript. JW, HG, CC, WeiZ, and L-YK performed the experiments. WenZ and JW analyzed the data. All authors reviewed the manuscript.

### Conflict of interest statement

The authors declare that the research was conducted in the absence of any commercial or financial relationships that could be construed as a potential conflict of interest.
